# A workflow for simplified analysis of ATAC-cap-seq data in R

**DOI:** 10.1093/gigascience/giy080

**Published:** 2018-06-28

**Authors:** Ram Krishna Shrestha, Pingtao Ding, Jonathan D G Jones, Dan MacLean

**Affiliations:** Sainsbury Laboratory, Norwich Research Park, Norwich, UK, NR4 7UH

**Keywords:** ATAC-seq, capture-seq, RNA-seq, genomics, R, workflows

## Abstract

**Background:**

Assay for Transposase-Accessible Chromatin (ATAC)-cap-seq is a high-throughput sequencing method that combines ATAC-seq with targeted nucleic acid enrichment of precipitated DNA fragments. There are increased analytical difficulties arising from working with a set of regions of interest that may be small in number and biologically dependent. Common statistical pipelines for RNA sequencing might be assumed to apply but can give misleading results on ATAC-cap-seq data. A tool is needed to allow a nonspecialist user to quickly and easily summarize data and apply sensible and effective normalization and analysis.

**Results:**

We developed atacR to allow a user to easily analyze their ATAC enrichment experiment. It provides comprehensive summary functions and diagnostic plots for studying enriched tag abundance. Application of between-sample normalization is made straightforward. Functions for normalizing based on user-defined control regions, whole library size, and regions selected from the least variable regions in a dataset are provided. Three methods for detecting differential abundance of tags from enriched methods are provided, including bootstrap *t*, Bayes factor, and a wrapped version of the standard exact test in the edgeR package. We compared the precision, recall, and *F*-score of each detection method on resampled datasets at varying replicate, significance threshold, and genes changed and found that the Bayes factor method had the greatest overall detection power, though edgeR was slightly stronger in simulations with lower numbers of genes changed.

**Conclusions:**

Our package allows a nonspecialist user to easily and effectively apply methods appropriate to the analysis of ATAC-cap-seq in a reproducible manner. The package is implemented in pure R and is fully interoperable with common workflows in Bioconductor.

## Introduction

Assay for Transposase-Accessible Chromatin (ATAC)-cap-seq can be conceptualized as a combination of two pre-existing, widely used methods; the high-throughput sequencing of DNA from targeted enrichment capture performed on DNA fragments obtained from prior ATAC [1]. ATAC-seq allows for rapid detection of accessible chromatin that may indicate open chromatin, DNA-binding protein binding sites, and nucleosome position. As ATAC-seq is fast and requires small amounts of input material [2], it is a popular and widely applicable assay used in a range of developmental [3, 4], medical [5, 6], environmental [7, 8], and technical studies [9]. Targeted sequence capture uses oligonucleotide baits to extract specific DNA fragments from a mixture and, when combined with ATAC-seq, allows an increase in sensitivity of detection and throughput for particular preselected genome regions at the expense of genome-wide detection. It is a trivial step to consider combining ATAC-seq and capture to use the advantages of each in a single experiment. However, doing so will raise new analytic concerns, which are discussed more fully below. ATAC-cap-seq does not show that chromatin is open in general, unless baits are tiled deliberately across continuous wide regions.

A typical ATAC-cap-seq may be done by beginning with an ATAC-seq library as described previously [2]. Next, small (approximately 9 nt) indexed sequence bar codes can be used to amplify the ATAC libraries. Fragments are size selected, e.g., using SageELF, to enrich sequences between 300 bp and 1.2 kb in order to give a uniform size distribution for multiplexing samples and replicates. Baits are designed and synthesized as 120-nt single-strand RNA baits covalently bound to biotinylated magnetic beads. These can be used in sequence capture with the multiplexed ATAC libraries. Libraries are quality checked and then sequenced. Capture-seq [10, 11] is a cost-effective alternative to expensive whole genome analysis. Scientists can focus on loci of interest and multiplex multiple samples and data types for the same sequencing cost as a single whole-genome sample.

Analysis of sequence reads from ATAC-seq begins with mapping and alignment to a genome followed by peak detection to identify read-enriched regions. A wide range of tools have been developed to perform peak finding, notably, MACS [12], HOMER [13], and SICER [14]. With these tools, the genome is divided into windows, and the read counts in those windows are analyzed. RNA sequencing (RNA-seq) packages that deal with read counts post-mapping work on estimates of read counts corresponding to regions that can be thought of as windows that represent genes or transcripts. The edgeR [15] and DESeq [16] packages implement negative binomial models to estimate differential counts between samples. The Bioconductor [17] package csaw uses fixed-width windows across the entire genome [18].

The enrichment capture step can produce a dataset with characteristics for which workflows designed for many thousands of windows may not give the best results. In particular, the number of regions represented in the target set may be small (tens rather than some thousands). Also, the selected regions in an enrichment capture experiment are likely to be related biologically and can conceivably covary as a small number or even a single unit. The count of each feature is also dependent on the magnitude of its abundance; the capture step results in overrepresentation of highly abundant features in the captured mixture. These unique features of ATAC-cap-seq data mean that normalization and differential count estimation must be applied carefully.

The tools and methods for solving this problem exist, but they have not been used together frequently in bioinformatics analysis, which has tended toward whole-genome, nonenriched sample analysis. Consequently, a nonspecialist user may find it difficult to bring useful methods together. Hence, a workflow that is based around these methods would prove useful to those beginning ATAC-cap-seq analysis from a nonspecialist background.

## Findings

A key aim of our atacR package is to allow the user to easily assess the success of their ATAC enrichment experiment and determine what further preparative work is required. It achieves this with comprehensive summaries and functions for diagnostic plots. Application between sample normalization is made straightforward. Functions to apply preselected control gene normalization, library size normalization, or normalization based on the least varying regions in the sample are implemented. Differential count estimation functions for the application of edgeR exact test, bootstrap *t* tests and a Bayes factor *t* test are provided. The package is implemented in pure R, its base objects are standard Bioconductor and, as such, is designed to be fully interoperable with common workflows in the Bioconductor framework.

### Workflow

The atacR workflow is based around three major steps: data loading and inspection, identification of best targets to use for normalization, and detection of differential count estimates. The package provides functions that make each step of the workflow straightforward and helps to make these potentially complex analyses more reproducible and the components re-useable in different contexts. Tutorial vignettes are provided that can be loaded directly from the R console.

#### Loading

The atacR package relies on Bioconductor SummarizedExperiment [19] container objects to record counts in user-defined windows. Window locations, binary alignment (BAM) file paths, and associated sample information are specified from general feature format files provided by the user. Read counts are loaded and calculated from BAM using the windowCounts method in R csaw [18] or Rsamtools [20]. A single function allows loading and read filtering directly from BAM files. The atacR package prepares these data into structures suitable for downstream analysis.

#### The atacr object

The atacr object describes sample metadata, bait locations, and the counts in target and nontarget windows. Generic summary and plot methods are available that quickly present diagnostic information from which the success of the experiment with respect to read alignment to on–off targets can swiftly be ascertained. Functions operating on this object each have a "by" parameter that allows the user to specify on–off target subsets to analyze. As the atacr object is essentially an R list, new data containing the counts after application of any processing step can be added to a custom slot and analyzed using atacr functions in the same syntax.

#### Diagnostic plots and normalizations

**Figure 1:**
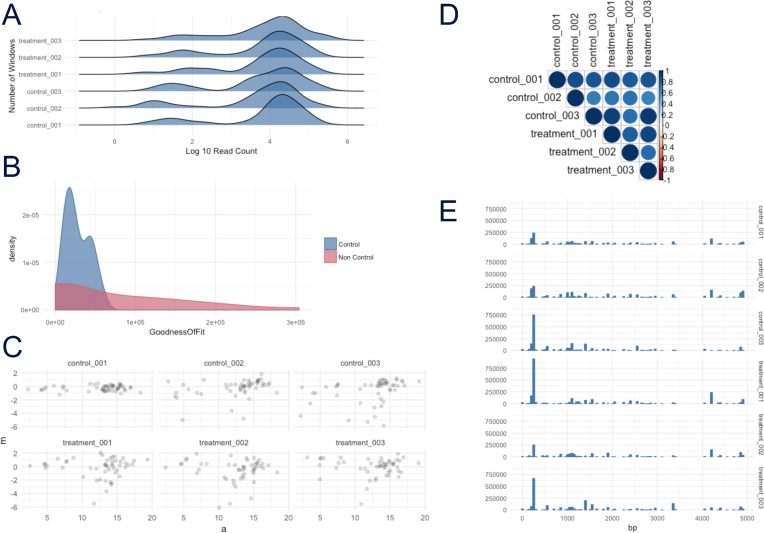
Example plots from atacR, generated on simulated data. **(A)** Per sample coverage count density. **(B)** GoF estimate density plot for control/noncontrol windows. **(C)** Per sample plot of log ratio versus average intensity (MA) plot. **(D)** Per sample similarity heat map. **(E)** Per sample chromosome coverage count histogram.

Data in the atacr object can be assessed for sample bias using specialized plot functions on a per sample and treatment basis. Plots can be generated using functions for whole sample count histograms, chromosome coverage density, Per sample plot of log ratio versus average intensity (MA) plots, heat maps comparing sample counts, density plots of genome regions' designated on–off target, and density plots of variability in regions nominated as normalization controls. See Fig.1 for examples.

atacR provides a small set of useful normalization methods applicable to small sets of target windows or those in which the large proportion show the same change in differential accessibility. A straightforward library size normalization is provided. For most ATAC purposes, this will be underpowered because the small number of windows or high proportions of changing windows will cause skew between samples. This method is useful when the experiment has reasonably high counts (>20 mean) and it is certain few windows (<10%) will display differential counts. The atacR package also implements a dynamic method based on estimating the goodness of fit (GoF) measure described in [21]. This method calculates GoF, a window/gene level measure of variability across all samples, and selects the windows with the lowest GoF as the subset on which to normalize. It is fast, automatically finds the least variation and the best features in the data to normalize with, and does a reasonable job of between-sample normalization. It is usually the best one to choose. It is particularly useful when it is not known whether many or just a few windows will be changing, as it should perform the same regardless. In addition to library size and GoF, a user-led method is provided in which control windows corresponding to regions of the genome not expected to show differential accessibility can be defined in a text file. This is passed to a normalization function that uses differences in these windows between samples or treatments to scale whole experiment counts. For ease of use with other normalization strategies, a set of custom normalization factors can also be provided as a simple vector and used directly.

#### Differential abundance and comparisons

The atacR package implements three methods of detecting differential abundance; the standard and effective edgeR method is wrapped for ease of use. A bootstrap *t* test and Bayes factor method are also provided. These can be run in a single factor manner on pairs of samples or on all samples simultaneously with a common reference sample specified by the user.

**Figure 2:**
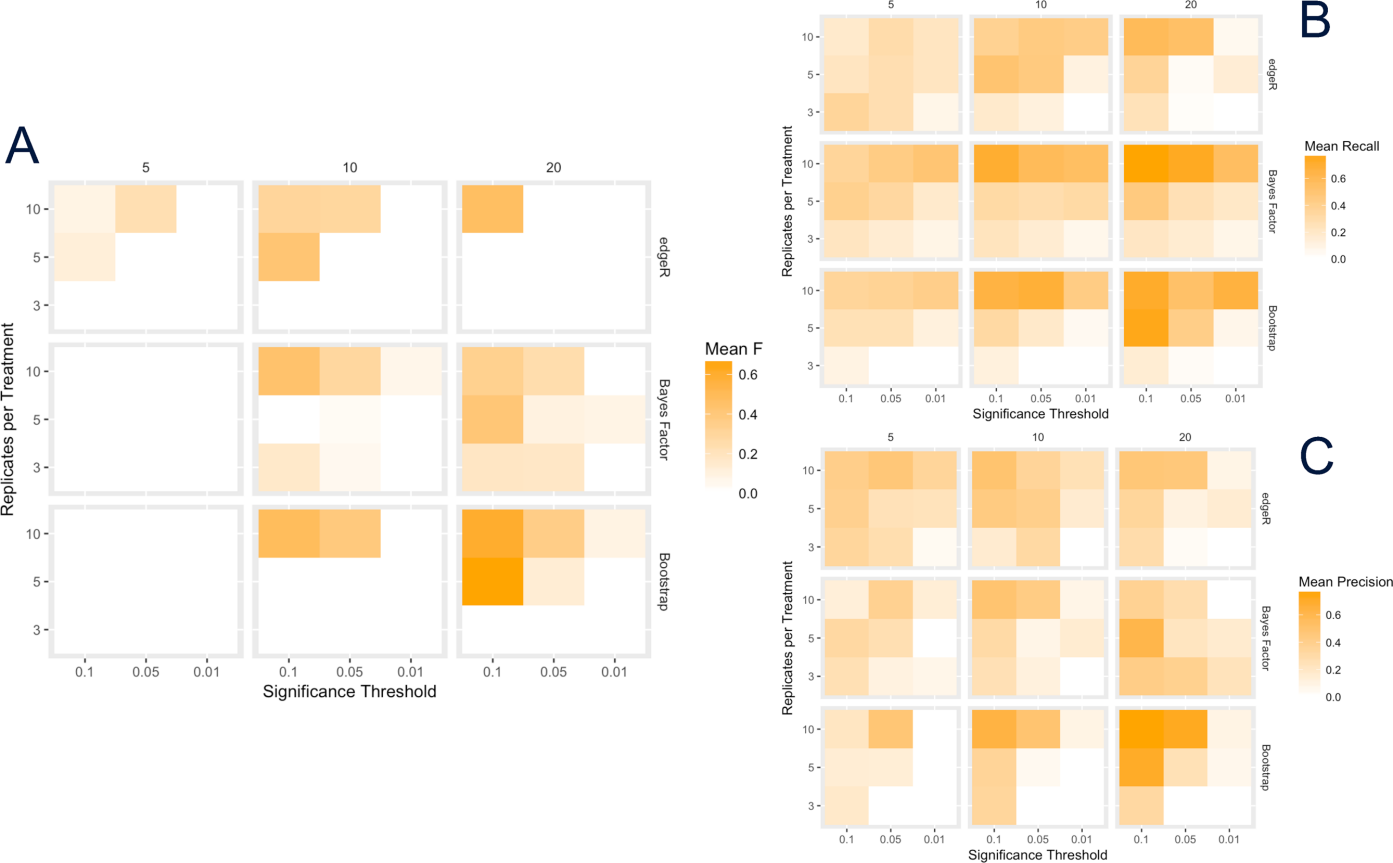
**(A)** Heat map of *F*-score, **(B)**recall, and **(C) p**recision for runs of edgeR exact test, bootstrap *t* test, and Bayes factor *t* test for varying sample replicate, significance threshold, and number of windows changed in simulated data.

We compared the precision, recall, and *F*-score of each method on simulated ATAC-cap-RNA-seq data at varying replicates, significance thresholds, and genes changed. To create a simulated dataset, we examined counts from three independent RNA-capseq datasets of 52 target-enriched regions. These showed a double peak in the count distribution, though the residual to the mean count was roughly normally distributed (Supplemental Information 1). We used the count set as a sample from which to randomly select base counts; from these, a preselected number was multiplied in all replicates of the treatment by a preselected factor to represent differential expression. Experimental noise was also simulated for each count. At each combination of parameters (Table1) The edgeR exact test, bootstrap *t* test, and Bayes factor methods in atacR were used to identify differentially abundant counts. We calculated precision, recall, and *F* as described in the Methods section. Ten iterations of the simulation were run and mean plotted (Fig.2B and 2C). The edgeR method performed best in recall and precision in all simulations with smaller numbers of changed windows (5), whereas bootstrap *t* and Bayes factor were stronger to recall at 10 and 20 changed windows. The bootstrap showed greatest precision at 20 changed windows. The *F*-score represents a balance between precision and recall. Here, we observed a slightly larger *F*-score Bayes factor over all parameter values tested when 20 windows were changed. The edgeR method had the highest *F*-scores when only five windows had differential counts. From this we conclude that Bayes factor is likely a good all-around method for data with many changing windows (in this experiment, approximately 40% of windows), whereas edgeR outperforms at lower levels (approximately 10%).

**Table 1:** Parameters for simulated datasets

Parameter	Values used
Replicates per treatment	3, 5, 10
Number of counts changed	5, 10, 20
Fold change	1, 5, 2, 4
Significance detection level	0.1, 0.05, 0.01*

* For Bayes factor runs, significant Bayes factor of 1.1, 1.5, and 2 were used.”

## Methods

To run simulations, 52 fake genome windows were defined in a control and treatment experiment. The counts for each window were selected from a dataset of 156 counts from a pilot wild-type Arabidopsis RNA-cap-seq experiment. These counts are stored in the atacR package as a data object "athal_wt_counts" for re-use. At each run of the simulation, the replicates per treatment, number of counts changed, fold ratio by which the counts changed, and the significance level at which detection was carried out were varied. For each combination of parameters described in Table1, we carried out 10 repetitions of the simulation. The edgeR exact test, bootstrap *t* test, and Bayes factor *t* test were performed on each run using atacR and counted as a true positive (TP), false positive (FP), or false negative. TP was defined as the number of windows set with differential counts that were correctly called by the detection method. FP was defined as the number of windows that were called but were not set with differential counts. FN was defined as the number of windows that were set as differential but were not called differential. From this precision, recall and *F* were calculated as follows:

(1) \begin{equation*}
Precision = \frac{TP}{TP + FP}
\end{equation*}

(2) \begin{equation*}
Recall = \frac{TP}{FN + TP}
\end{equation*}

(3) \begin{equation*}
F = 2 \frac{precision \times recall}{precision + recall}
\end{equation*}

The simulated data experiments were carried out in RStudio. The entire experiment code is provided in the Supplemental Materials. These are executable RMD files that can be rerun to reproduce our experiment exactly in the R programming language.

The version of atacR used was 0.4.13. The base counts that were modified in simulations are available in the atacR package in the object “atacr::athal_wt_counts.”

Simulations and analyses were run on an Apple Macintosh computer with R and OS specifications as described in Table 2

**Table 2:** Machine used to run analyses

Environment parameter	Values
platform	x86_64-apple-darwin15.6.0
arch	x86_64
os	darwin15.6.0
system	x86_64, darwin15.6.0
major	3
minor	4.2
year	2017
month	09
day	28
svn rev	73368
language	R
version.string	R version 3.4.2 (2017-09-28)
nickname	Short Summer

## Availability of source code

- Project name: atacR
- Project home page: https://github.com/TeamMacLean/atacr
- Operating system(s): Platform independent
- Programming language: R
- License: GNU GPL 3

The library is provided as an R package that can be installed from Github using devtools::install_from_github(’TeamMacLean/atacr’).

## Supplementary Material

GIGA-D-18-00020_Original_Submission.pdf

GIGA-D-18-00020_Revision_1.pdf

GIGA-D-18-00020_Revision_2.pdf

GIGA-D-18-00020_Revision_3.pdf

Response_to_Reviewer_Comments_Original_Submission.pdf

Response_to_Reviewer_Comments_Revision_1.pdf

Response_to_Reviewer_Comments_Revision_2.pdf

Reviewer_1_Report_(Original_Submission) -- Noboru Jo Sakabe 1/29/2018 Reviewed

Reviewer_1_Report_(Revision_1) -- Noboru Jo Sakabe 5/23/2018 Reviewed

Reviewer_2_Report_(Original_Submission) -- James Lewis 3/15/2018 Reviewed

Additional Files

## Availability of supporting data

The R code supporting the results presented here is available in the repository (https://github.com/TeamMacLean/atacr). The software is registered in the SciCrunch.org database with a Research Resource Identification Initiative ID of SCR_016286.

An archival copy of the code and results of resampling experiments are also available via the *GigaScience* repository GigaDB [22].

## Additional files

Simulations.csv

001_methods_comparison.Rmd

002_methods_comparison.Rmd

## Abbreviations

ATAC: Assay for Transposase-Accessible Chromatin; BAM: binary alignment; FN: false negative; FP: false positive; GoF: goodness of Fit; RNA-seq: RNA sequencing; TP: true positive.
