# Supplementary material for: A workflow for simplified analysis of ATAC-cap-seq data in R
Source: Gigascience. 2018 Jun 28;7(7):giy080. doi: 10.1093/gigascience/giy080 (PMC6047409; doi:10.1093/gigascience/giy080)
Supplement: GIGA-D-18-00020_Revision_3.pdf [file giy080_giga-d-18-00020_revision_3.pdf]

GigaScience

A workflow for simplified analysis of ATAC-cap-seq data in R

--Manuscript Draft--

**Manuscript Number:**	GIGA-D-18-00020R3
**Full Title:**	A workflow for simplified analysis of ATAC-cap-seq data in R
**Article Type:**	Technical Note
**Funding Information:**	H2020 Marie Skłodowska-Curie Actions (656243) — Dr Pingtao Ding
**Abstract:**	ATAC-cap-seq is a high-throughput sequencing method that combines targeted nucleic acid enrichment of precipitated DNA fragments with an upstream ATAC-seq step. There are increased analytical difficulties arising from working with a set of regions of interest that may be small in number and biologically dependent. Common statistical pipelines for RNAseq might be assumed to apply but can give misleading results on ATAC-cap-seq data. A tool is needed to allow a non-specialist user to quickly and easily summarise data and apply sensible and effective normalisation and analysis.<br><br>We developed atacR to allow a user to easily analyse their ATAC enrichment experiment. It provides comprehensive summary functions and diagnostic plots for studying enriched tag abundance. Applying between-sample normalisation is made straightforward and functions for normalising based on user-defined control regions, whole library size and regions selected from the least variable regions in a dataset are provided. Three methods for detecting differential abundance of tags from enriched methods are provided, including Bootstrap t, Bayes Factor and a wrapped version of the standard exact test in the edgeR package. We compared the precision, recall and F-score of each detection method on resampled datasets at varying replicate, significance threshold and genes changed, we found that the Bayes factor method had greatest overall detection power, though edgeR was slightly stronger in simulations with lower numbers of genes changed.<br><br>Our package allows a non-specialist user to easily and effectively apply methods appropriate to the analysis of ATAC-cap-seq in a reproducible manner. The package is implemented in pure R and is fully interoperable with common workflows in Bioconductor.
**Corresponding Author:**	Dan MacLean<br><br>UNITED KINGDOM
**Corresponding Author Secondary Information:**	
**Corresponding Author's Institution:**	
**Corresponding Author's Secondary Institution:**	
**First Author:**	Ram Krishna Shrestha, PhD
**First Author Secondary Information:**	
**Order of Authors:**	Ram Krishna Shrestha, PhD
	Pingtao Ding, PhD
	Jonathan DG Jones, PhD
	Dan MacLean
**Order of Authors Secondary Information:**	
**Response to Reviewers:**	None.
**Additional Information:**	
**Question**	**Response**

Powered by Editorial Manager® and ProduXion Manager® from Aries Systems Corporation

Are you submitting this manuscript to a special series or article collection?	No
**Experimental design and statistics**<br><br>Full details of the experimental design and statistical methods used should be given in the Methods section, as detailed in our Minimum Standards Reporting Checklist. Information essential to interpreting the data presented should be made available in the figure legends.<br><br>Have you included all the information requested in your manuscript?	Yes
**Resources**<br><br>A description of all resources used, including antibodies, cell lines, animals and software tools, with enough information to allow them to be uniquely identified, should be included in the Methods section. Authors are strongly encouraged to cite Research Resource Identifiers (RRIDs) for antibodies, model organisms and tools, where possible.<br><br>Have you included the information requested as detailed in our Minimum Standards Reporting Checklist?	Yes
**Availability of data and materials**<br><br>All datasets and code on which the conclusions of the paper rely must be either included in your submission or deposited in publicly available repositories (where available and ethically appropriate), referencing such data using a unique identifier in the references and in the "Availability of Data and Materials" section of your manuscript.<br><br>Have you have met the above requirement as detailed in our Minimum Standards Reporting Checklist?	Yes

Powered by Editorial Manager® and ProduXion Manager® from Aries Systems Corporation

Manuscript

Click here to view linked References

Click here to access/download;Manuscript;workflow-simplified-analysis.pdf

OXFORD

(GIGA)^n SCIENCE

GigaScience, 2017, 1–6

doi: xx.xxxx/xxxx
Manuscript in Preparation
Paper

PAPER

A workflow for simplified analysis of ATAC-cap-seq data in R

Ram Krishna Shrestha[1, †], Pingtao Ding[1, †], Jonathan DG Jones[1] and Dan MacLean[1,*]

[1]The Sainsbury Laboratory, Norwich Research Park, Norwich, UK, NR4 7UH

[†] These authors contributed equally
[*] To whom correspondence should be addressed
[†]ram-krishna.shrestha@tsl.ac.uk
[†]pingtao.ding@tsl.ac.uk
jonathan.jones@tsl.ac.uk
[*]dan.maclean@tsl.ac.uk

Abstract

Background ATAC-cap-seq is a high-throughput sequencing method that combines ATAC-seq with targeted nucleic acid enrichment of precipitated DNA fragment. There are increased analytical difficulties arising from working with a set of regions of interest that may be small in number and biologically dependent. Common statistical pipelines for RNAseq might be assumed to apply but can give misleading results on ATAC-cap-seq data. A tool is needed to allow a non-specialist user to quickly and easily summarise data and apply sensible and effective normalisation and analysis. **Results** We developed atacR to allow a user to easily analyse their ATAC enrichment experiment. It provides comprehensive summary functions and diagnostic plots for studying enriched tag abundance. Applying between-sample normalisation is made straightforward and functions for normalising based on user-defined control regions, whole library size and regions selected from the least variable regions in a dataset are provided. Three methods for detecting differential abundance of tags from enriched methods are provided, including Bootstrap *t*, Bayes Factor and a wrapped version of the standard exact test in the edgeR package. We compared the precision, recall and F-score of each detection method on resampled datasets at varying replicate, significance threshold and genes changed, we found that the Bayes factor method had greatest overall detection power, though edgeR was slightly stronger in simulations with lower numbers of genes changed. **Conclusions** Our package allows a non-specialist user to easily and effectively apply methods appropriate to the analysis of ATAC-cap-seq in a reproducible manner. The package is implemented in pure R and is fully interoperable with common workflows in Bioconductor.

Key words: ATAC-seq; capture-seq; RNAseq; genomics; R; workflows;

Introduction

ATAC-cap-seq can be conceptualised as a combination of two pre-existing, widely-used methods: the high-throughput sequencing of DNA from targeted enrichment capture performed on DNA fragments obtained from prior Assay for Transposase-Accessible Chromatin (ATAC)[1]. ATAC-seq allows for rapid detection of accessible chromatin that may indicate open chromatin, DNA-binding protein binding sites and nucleosome position. As ATAC-seq is fast and requires low amounts of input material [2] it is a popular and widely applicable assay used in a range of developmental [3, 4] , medical [5, 6], environmental [7, 8] and technical studies [9]. Targeted sequence capture uses oligonucleotide baits to extract specific DNA fragments from a

Compiled on: June 20, 2018.
Draft manuscript prepared by the author.

mixture and when combined with ATAC-seq allows an increase in sensitivity of detection and throughput for particular preselected genome regions at the expense of genome wide detection. It is a trivial step to consider combining ATAC-seq and capture to use the advantages of each in a single experiment. However, doing so will raise new analytic concerns, discussed more fully below. ATAC-cap-seq does not show that chromatin is open in general, unless baits are tiled deliberately across continuous wide regions.

A typical ATAC-cap-seq may be done by beginning with an ATAC-seq library as described previously [2]. Next, small (approximately 9 nt) indexed sequence barcodes can be used to amplify the ATAC libraries, Fragments are size selected, e.g. using SageELF to enrich sequences between 300bp and 1.2kb to give a uniform size distribution for multiplexing samples and replicates. Baits are designed and synthesised as 120 nt single-strand RNA baits covalently bound to biotinylated magnetic beads. These can be used in sequence capture with the multiplexed ATAC libraries. Libraries are quality checked then sequenced. Capture-seq [10, 11] is a cost-effective alternative to expensive whole genome analysis. Scientists can focus on loci of interest and multiplex multiple samples and data types for the same sequencing cost as a single whole genome sample.

Analysis of sequence reads from ATAC-seq begins with mapping and alignment to a genome followed by peak detection to identify read enriched regions. A wide range of tools have been developed to perform peak finding, notably MACS [12], HOMER [13] and SICER [14]. In these the genome is divided into windows and the read counts in those analysed. RNAseq packages that deal with read counts post-mapping work on estimates of read counts corresponding to regions that can be thought of as windows that represent genes or transcripts. The edgeR [15] and DESeq [16] packages implement Negative Binomial models to estimate differential counts between samples. The Bioconductor [17] package csaw uses fixed width windows across the entire genome [18].

The enrichment capture step can produce a data set with characteristics that mean workflows designed for many thousands of windows may not give best results. In particular the number of regions represented in the target set may be small (many tens rather than some thousands). Also the selected regions in an enrichment capture experiment are likely to be related biologically and can conceivably co-vary as a small number or even a single unit. The count of each feature is also dependent on the magnitude of its abundance, the capture step results in over-representation of highly abundant features in the captured mixture. These unique features of ATAC-cap-seq data mean that normalisation and differential count estimation must be applied carefully.

The tools and methods for solving this problem already exist, but they have not been used together frequently in bioinformatics analysis, which have tended toward whole genome, non-enriched sample analysis. Consequently a non-specialist user may find it difficult to bring useful methods together. Hence a workflow that is based around these methods would prove useful to those beginning ATAC-cap-seq analysis from a non-specialist background.

Findings

A key aim of our atacR package is to allow the user to easily assess the success of their ATAC enrichment experiment and determine what further preparative work is required. It achieves this with comprehensive summaries and functions for diagnostic plots. Applying between sample normalisation is made straightforward. Functions to apply pre-selected control gene normalisation, library size normalisation or normalisation based on the least varying regions in the sample are implemented. Differential count estimation functions for the application of edgeR exact-test, bootstrap *t*-tests and a Bayes factor *t*-test are provided. The package is implemented in pure R, it's base objects are standard Bioconductor and as such is designed to be fully interoperable with common workflows in the Bioconductor framework.

Workflow

The atacR workflow is based around three major steps - data loading and inspection, identification of best targets to use for normalisation and detection of differential count estimates. The package provides functions that make each step of the workflow straightforward and helps to make these potentially complex analyses more reproducible and the components reuseable in different contexts. Tutorial vignettes are provided that can be loaded directly from the R console.

Loading

The atacR package relies on Bioconductor SummarizedExperiment [19] container objects to record counts in user defined windows. Window locations, BAM file paths and associated sample information are specified from GFF files provided by the user. Read counts are loaded and calculated from BAM using the windowCounts method in R csaw [18] or Rsamtools [20]. A single function allows loading and read filtering directly from BAM files. The atacR package prepares these data into structures suitable for downstream analysis.

The atacr object

The atacr object describes sample metadata, bait locations and the counts in target and non-target windows. Generic summary and plot methods are available that quickly present diagnostic information from which the success of the experiment with respect to read alignment to on/off targets can swiftly be ascertained. Functions operating on this object each have a 'by' parameter which allows the user to specify on/off target subsets to analyse. As the atacr object is essentially an R list, new data containing the counts after application of any processing step can be added to a custom slot and analysed using atacr functions in the same syntax.

Diagnostic plots and normalisations

Data in the atacr object can be assessed for sample bias using specialised plot functions on a per sample and treatment basis, Plots can be generated using functions for whole sample count histograms, chromosome coverage density, MA plots, heatmaps comparing sample counts, density plots of genome regions designated on/off target and density plots of variability in regions nominated as normalisation controls. See Figure 1 for examples.

atacR provides a small set of useful normalisation methods applicable to small sets of target windows or those in which the large proportion show the same change in differential accessibility. A straightforward library size normalisation is provided. For most ATAC purposes this will be underpowered, because the low number of windows or high proportions of changing windows will cause skew between samples. This method useful when the experiment has reasonably high counts (> 20 mean) and it is certain few windows (< 10%) will display differential counts. The atacR package also implements a dynamic method based on estimating the Goodness of Fit (GoF) measure described in [21]. This method calculates GoF, a window/gene level measure of variability across all samples and selects the windows with lowest GoF as the subset on which to normalise. It is fast, automatically finds the least varying and best fea-

Table 1. Parameters for simulated datasets

Parameters	Values Used
Replicates per treatment	3,5,10
Number of counts changed	5, 10, 20
Fold change	1,5, 2, 4
Significance detection level	0.1, 0.05, 0.01*

*For Bayes Factor runs, significance levels were Bayes Factor of 1.1, 1.5 and 2 were used.

tures in the data to normalise with and does a reasonable job of between-sample normalisation. It is usually the best one to choose. It is particularly useful when it is not known whether many windows will be changing or just a few will be, as it should perform the same regardless. Further to library size and GoF a user-led method is provided in which control windows corresponding to regions of the genome not expected to show differential accessibility can be defined in a text file. This is passed to a normalisation function that uses differences in these windows between samples or treatments to scale whole experiment counts. For ease of use with other normalisation strategies, a set of custom normalisation factors can also be provided as a simple vector and used directly.

Differential abundance and comparisons

The atacR package implements three methods of detecting differential abundance; the standard and effective edgeR method is wrapped for ease- of-use. A bootstrap-*t* test and Bayes factor method are also provided. These can be run in single factor manner on pairs of samples, or on all samples simultaneously with a common reference sample specified by the user.

We compared the precision, recall and *F*-score of each method on simulated ATAC-cap-RNAseq data at varying replicate, significance threshold and genes changed. To create a simulated dataset we examined counts from three independent RNA-capseq datasets of 52 target enriched regions. These showed a double peak in the count distribution, though the residual to the mean count was roughly normally distributed (Supplemental Information 1). We used the count set as a sample from which to randomly select base counts and from these a preselected number were multiplied in all replicates of the treatment by a preselected factor to represent differential expression. Experimental noise was also simulated for each count. At each combination of parameters (Table 1) The edgeR exact-test, Bootstrap *t* test and Bayes Factor methods in atacR were used to identify differentially abundant counts. We calculated precision, recall and *F* as described in methods. Ten iterations of the simulation were run and mean plotted in Figure 2 B and C. The edgeR method performed best in recall and precision in all simulations with lower numbers of changed windows (5) whereas Bootstrap *t* and Bayes Factor were stronger to recall at 10 and 20 changed windows. The Bootstrap showed greatest precision at 20 changed windows. The *F*-score represents a balance between precision and recall, here we observed slightly larger *F*-score Bayes Factor over all parameters values tested when 20 windows were changed. The edgeR method had highest *F*-scores when only five windows had differential counts. From this we conclude that Bayes Factor is a likely good all round method in data with many changing windows (in this experiment approximately 40 percent of windows), whereas edgeR out-performs at lower levels (approximately ten percent).

Table 2. Machine used to run analyses.

Environment Parameters	Values
platform	x86_64-apple-darwin15.6.0
arch	x86_64
os	darwin15.6.0
system	x86_64, darwin15.6.0
major	3
minor	4.2
year	2017
month	09
day	28
svn rev	73368
language	R
version.string	R version 3.4.2 (2017-09-28)
nickname	Short Summer

Methods

To run simulations, 52 fake genome windows were defined in a control and treatment experiment. The counts for each window were selected from a dataset of 156 counts from a pilot wild-type Arabidopsis RNAcap-seq experiment. These counts are stored in the atacR package as a data object 'athal_wt_counts' for re-use. At each run of the simulation the replicates per treatment, number of counts changed, the fold ratio by which the counts change and the significance level at which detection was carried out was varied. For each combination of parameters described in Table 1 we carried out ten repetitions of the simulation. The edgeR exact-test, Bootstrap *t* test and Bayes Factor *t* test were performed on each run using atacR and counted True Positive (TP) False Positive (FP) and False Negatives. TP was defined as the number of windows set with differential counts that were correctly called by the detection method. FP was defined as the number of windows that were called but were not set with differential counts. FN is the number of windows that were set as differential but were not called differential. From these precision, recall and *F* were calculated as below.

$$Precision = \frac{TP}{TP + FP} \tag{1}$$

$$Recall = \frac{TP}{FN + TP} \tag{2}$$

$$F = 2\frac{precision \times recall}{precision + recall} \tag{3}$$

The simulated data experiments were carried out in RStudio. The whole experiment code is provided in Supplemental Materials. These are executable RMD files that can be re-run to reproduce our experiment exactly in the R programming language.

The version of atacR used was 0.4.13. The base counts that were modified in simulations are available in the atacR package in the object 'atacr::athal_wt_counts'

Simulations and analyses were run on an Apple Macintosh computer with R and OS specifications as described in Table 2

Availability of source code and requirements

- Project name: atacR
- Project home page: `https://github.com/TeamMacLean/atacr`
- Operating system(s): Platform independent

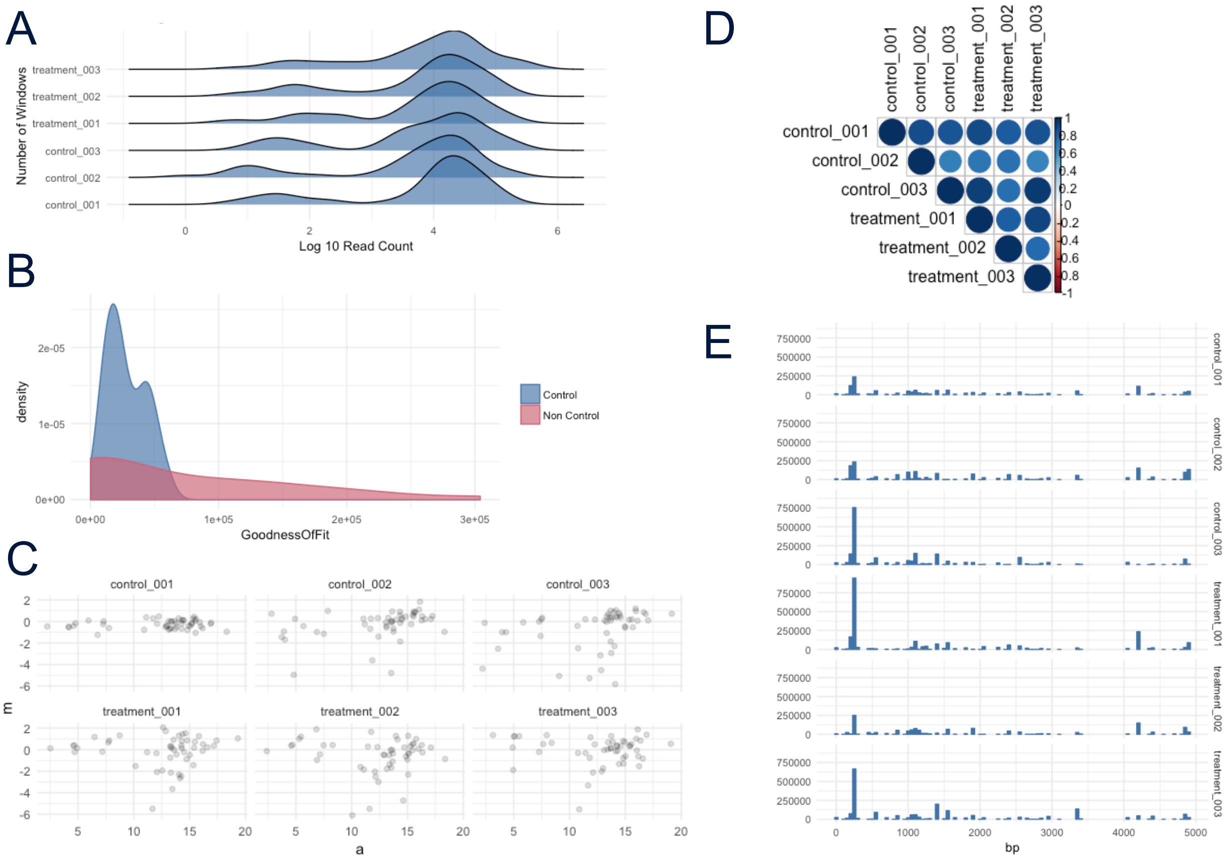

Figure 1. Example plots from atacR, generated on simulated data. A. Per sample coverage count density, B. GoF estimate density plot for control / non-control windows. C. Per sample MA plot. D. Per sample similarity heatmap. E. Per sample chromosome coverage count histogram

- Programming language: R
- License: GNU GPL 3

The library is provided as an R package that can be installed from Github using devtools::install_from_github('TeamMacLean/atacr')

Availability of supporting data and materials

The R code supporting the results of this article is available in the [https://github.com/TeamMacLean/atacr] repository. The software is registered in the SciCrunch.org database with a Research Resource Identification Initiative ID of SCR_016286.

An archival copy of the code and results of resampling experiments are also available via the GigaScience repository GigaDB [22]

Declarations

List of abbreviations

GoF - Goodness of Fit TP - True positive FP - False positive FN - False negative

Competing Interests

The authors declare that they have no competing interests.

Funding

RS, JDGJ and DM were supported by The Gatsby Charitable Foundation. This project received funding from the European Union's Horizon 2020 research and innovation programme under the Marie Skłodowska-Curie grant agreement No. 656243 to PD.

Author's Contributions

Conceptualization - DM and PD; Methodology - DM; Software - DM and RS; Formal Analysis - DM; Investigation - DM; Resources - PD; Data Curation RKS and DM; Writing - DM and PD; Visualization - DM and RS; Supervision - JDGJ; Project Administration - JDGJ; Funding Acquisition PD and JDGJ;

Acknowledgements

Thanks to Richard Morris of The John Innes Centre for very helpful discussion regarding Bayesian statistics.

References

1. Buenrostro JD, Giresi PG, Zaba LC, Chang HY, Greenleaf WJ. Transposition of native chromatin for fast and sensitive epigenomic profiling of open chromatin, DNA-binding proteins and nucleosome position. Nature methods 2013 Dec;10(12):1213–1218.
2. Buenrostro JD, Wu B, Chang HY, Greenleaf WJ. ATAC-

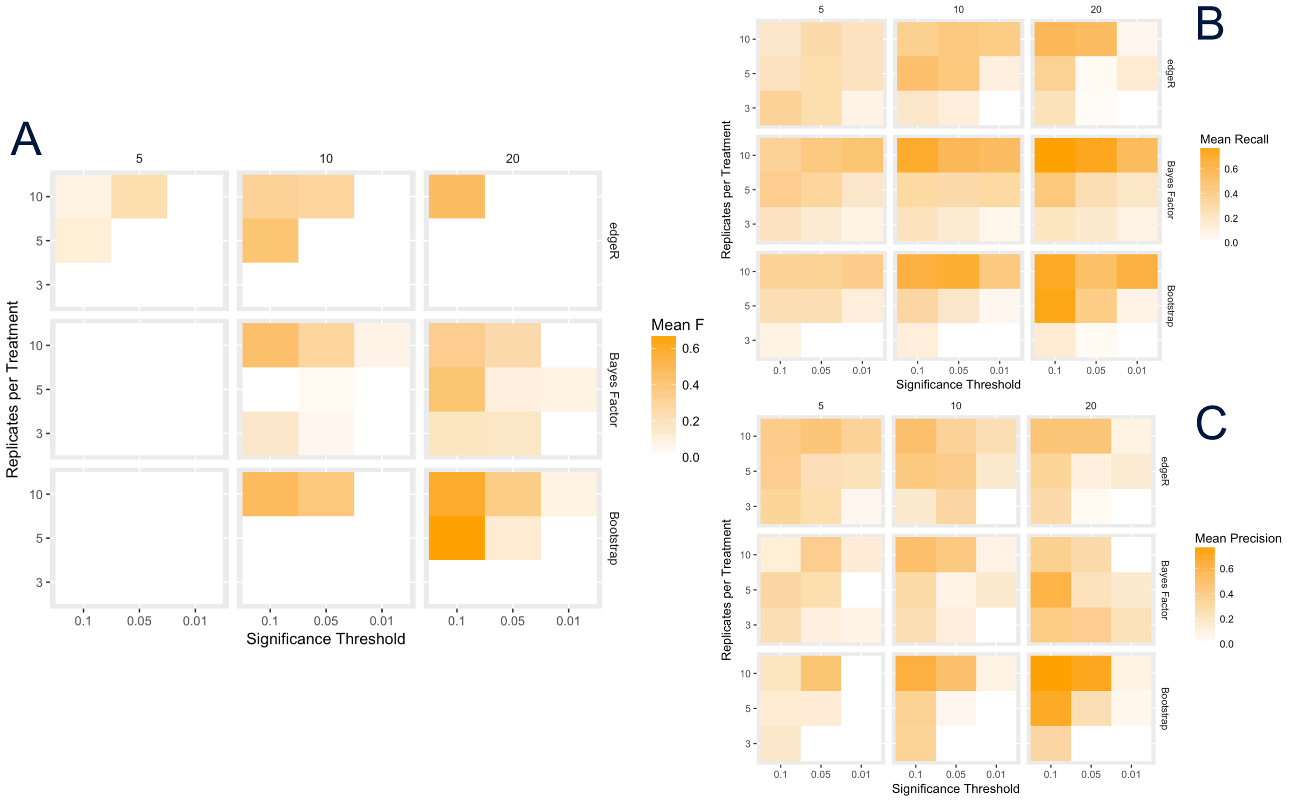

Figure 2. Heatmap of *F*-score (A), Recall (B) and Precision (C) for runs of edgeR exact-test, Bootstrap *t*-test or Bayes Factor *t* test for varying sample replicate, significance threshold and number of windows changed in simulated data

seq: A Method for Assaying Chromatin Accessibility Genome-Wide. Current protocols in molecular biology 2015 Jan;109:21.29.1–9.

3. De Kumar B, Parker HJ, Parrish ME, Lange JJ, Slaughter BD, Unruh JR, et al. Dynamic regulation of Nanog and stem cell-signaling pathways by Hoxa1 during early neuro-ectodermal differentiation of ES cells. ProcNatlAcadSciUSA 2017 Jun;114(23):5838–5845.
4. Whittaker DE, Riegman KLH, Kasah S, Mohan C, Yu T, Sala BP, et al. The chromatin remodeling factor CHD7 controls cerebellar development by regulating reelin expression. The Journal of clinical investigation 2017 Mar;127(3):874–887.
5. Garcia E, Hayden A, Birts C, Britton E, Cowie A, Pickard K, et al. Authentication and characterisation of a new oesophageal adenocarcinoma cell line: MFD-1. Scientific reports 2016 Sep;6:32417.
6. Litzenburger UM, Buenrostro JD, Wu B, Shen Y, Sheffield NC, Kathiria A, et al. Single-cell epigenomic variability reveals functional cancer heterogeneity. Genome biology 2017 Jan;18(1):15.
7. Song L, Huang SSC, Wise A, Castanon R, Nery JR, Chen H, et al. A transcription factor hierarchy defines an environmental stress response network. Science (New York, NY) 2016 Nov;354(6312).
8. Wilkins O, Hafemeister C, Plessis A, Holloway-Phillips MM, Pham GM, Nicotra AB, et al. EGRINs (Environmental Gene Regulatory Influence Networks) in Rice That Function in the Response to Water Deficit, High Temperature, and Agricultural Environments. The Plant cell 2016 Oct;28(10):2365–2384.
9. Montefiori L, Hernandez L, Zhang Z, Gilad Y, Ober C, Crawford G, et al. Reducing mitochondrial reads in ATAC-seq using CRISPR/Cas9. Scientific Reports 2017 May;7(1):2451.
10. Ng SB, Turner EH, Robertson PD, Flygare SD, Bigham AW, Lee C, et al. Targeted Capture and Massively Parallel Sequencing of Twelve Human Exomes. Nature 2009 Sep;461(7261):272–276. https://www.ncbi.nlm.nih.gov/pmc/articles/PMC2844771/.
11. Jupe F, Witek K, Verweij W, Sliwka J, Pritchard L, Etherington GJ, et al. Resistance gene enrichment sequencing (RenSeq) enables reannotation of the NB-LRR gene family from sequenced plant genomes and rapid mapping of resistance loci in segregating populations. The Plant Journal 2013;76(3):530–544. http://onlinelibrary.wiley.com/doi/10.1111/tpj.12307/abstract.
12. Zhang Y, Liu T, Meyer CA, Eeckhoute J, Johnson DS, Bernstein BE, et al. Model-based analysis of ChIP-Seq (MACS). Genome biology 2008;9(9):R137.
13. Heinz S, Benner C, Spann N, Bertolino E, Lin YC, Laslo P, et al. Simple combinations of lineage-determining transcription factors prime cis-regulatory elements required for macrophage and B cell identities. Molecular cell 2010 May;38(4):576–589.
14. Zang C, Schones DE, Zeng C, Cui K, Zhao K, Peng W. A clustering approach for identification of enriched domains from histone modification ChIP-Seq data. Bioinformatics (Oxford, England) 2009 Aug;25(15):1952–1958.
15. McCarthy, J D, Chen, Yunshun, Smyth, K G. Differential expression analysis of multifactor RNA-Seq experiments with respect to biological variation. Nucleic Acids Research 2012;40(10):–9.

16. Anders S, Huber W. Differential expression analysis for sequence count data. Genome Biology 2010;11:R106. http://genomebiology.com/2010/11/10/R106/.
17. Huber, W , Carey, J V, Gentleman, R , et al. Orchestrating high-throughput genomic analysis with Bioconductor. Nature Methods 2015;12(2):115–121. http://www.nature.com/nmeth/journal/v12/n2/full/nmeth.3252.html.
18. Lun ATL, Smyth GK. csaw: a Bioconductor package for differential binding analysis of ChIP-seq data using sliding windows. Nucleic Acids Res 2016;44(5):e45.
19. Morgan M, Obenchain V, Hester J, Pagès H. SummarizedExperiment: SummarizedExperiment container; 2017, r package version 1.6.3.
20. Morgan M, Pagès H, Obenchain V, Hayden N. Rsamtools: Binary alignment (BAM), FASTA, variant call (BCF), and tabix file import; 2017, http://bioconductor.org/packages/release/bioc/html/Rsamtools.html, r package version 1.28.0.
21. Li J, Witten DM, Johnstone IM, Tibshirani R. Normalization, testing, and false discovery rate estimation for RNA-sequencing data. Biostatistics 2012;13(3):523–538. +http://dx.doi.org/10.1093/biostatistics/kxr031.
22. Shrestha R, Ding P, Jones J, MacLean D. Supporting data for "A workflow for simplified analysis of ATAC-cap-seq data in R". GigaScience Database 2018;http://dx.doi.org/10.5524/100476.

Click here to access/download;Figure;heatmaps.pdf

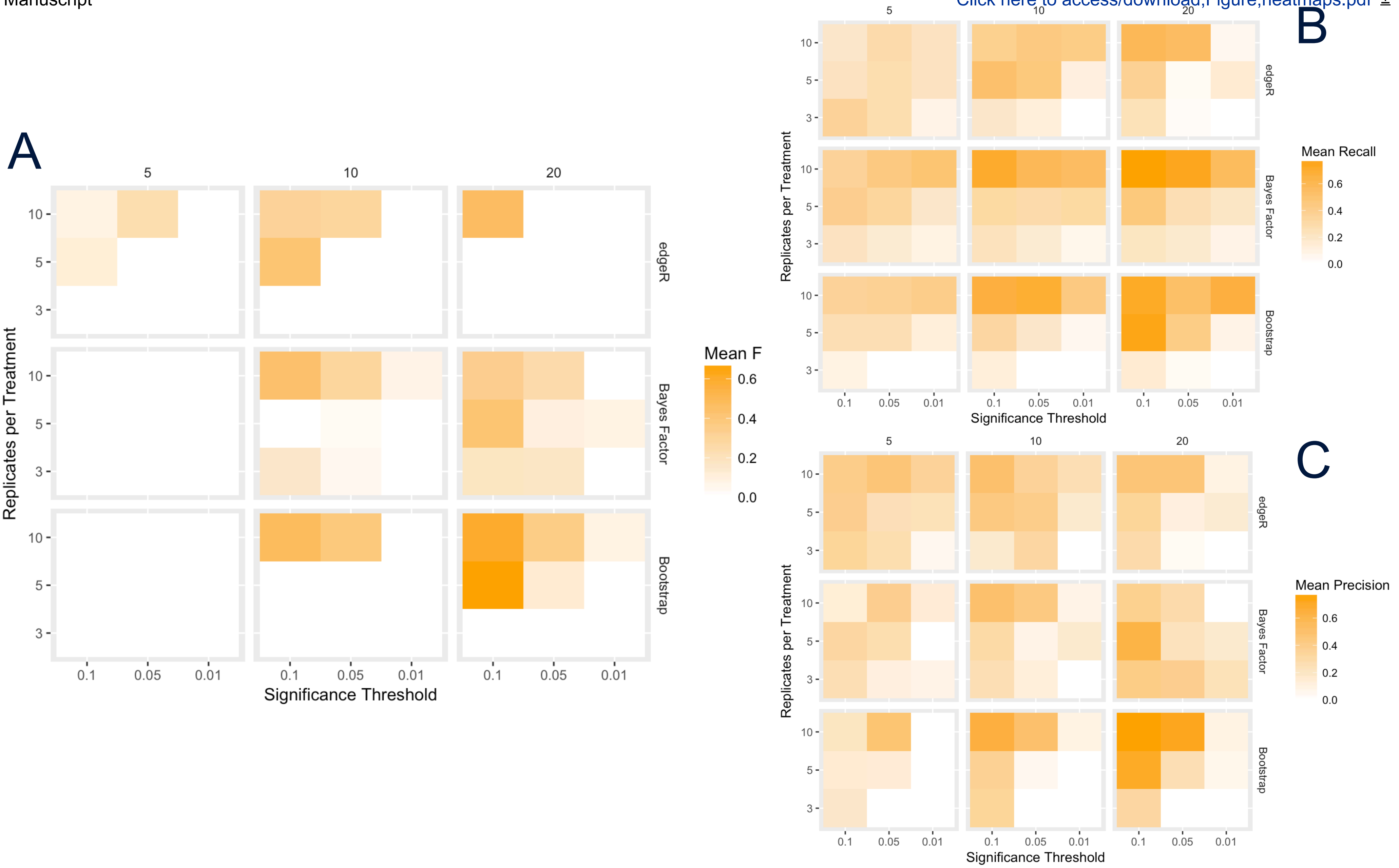

Manuscript
Click here to access/download;Figure;heatmaps.png

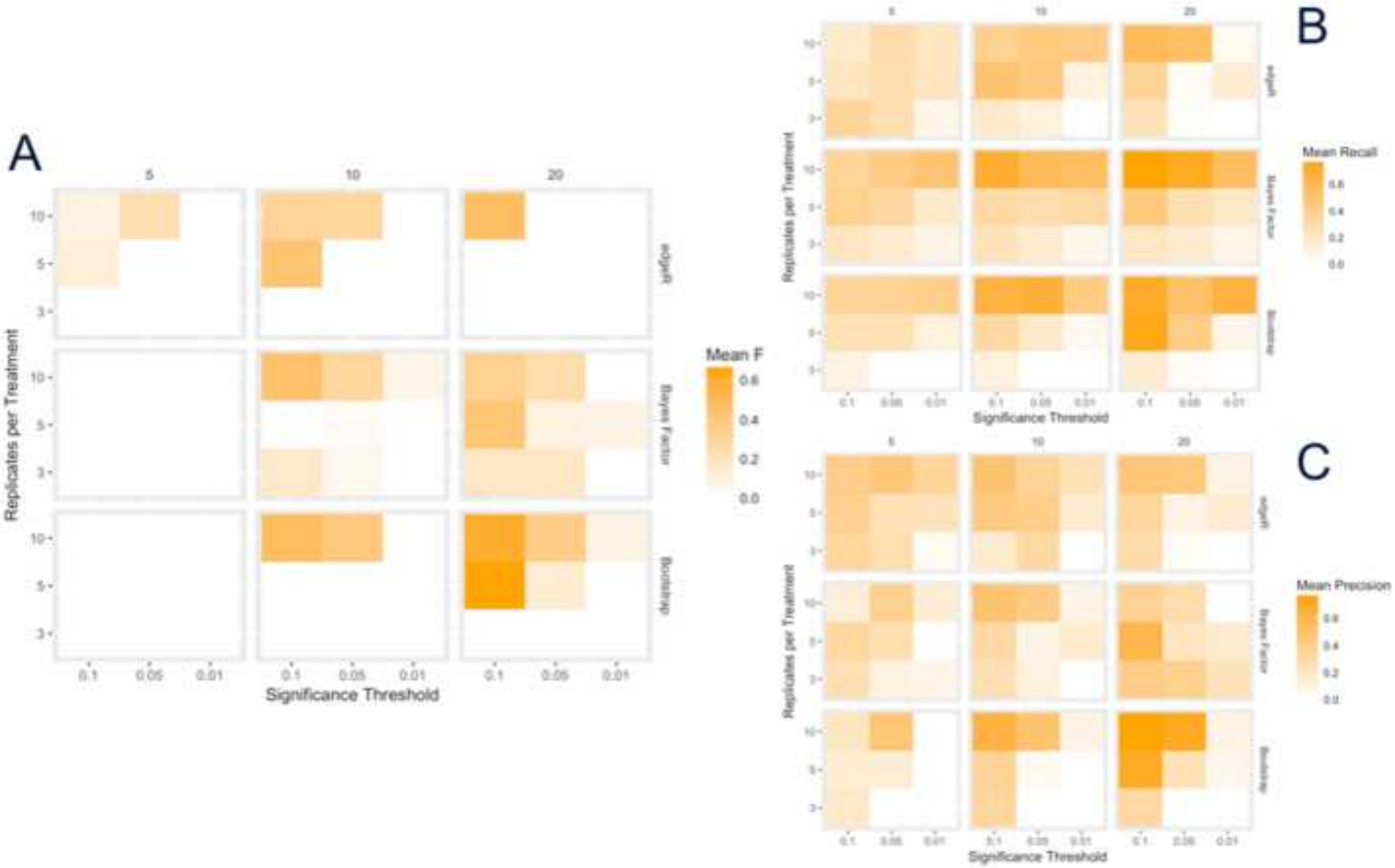

Manuscript

Click here to access/download;Figure;plots.pdf

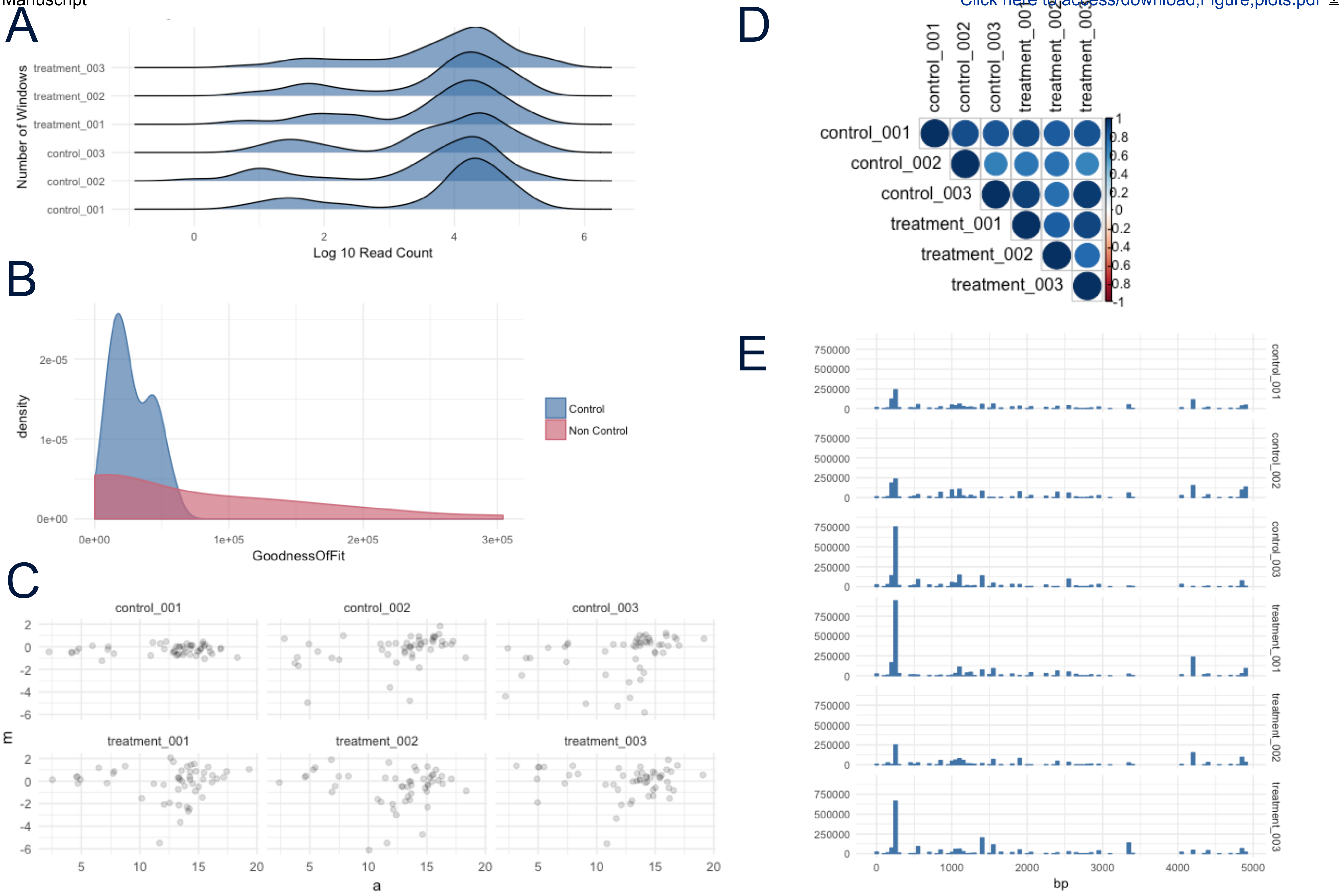

Manuscript
Click here to access/download;Figure;plots.png

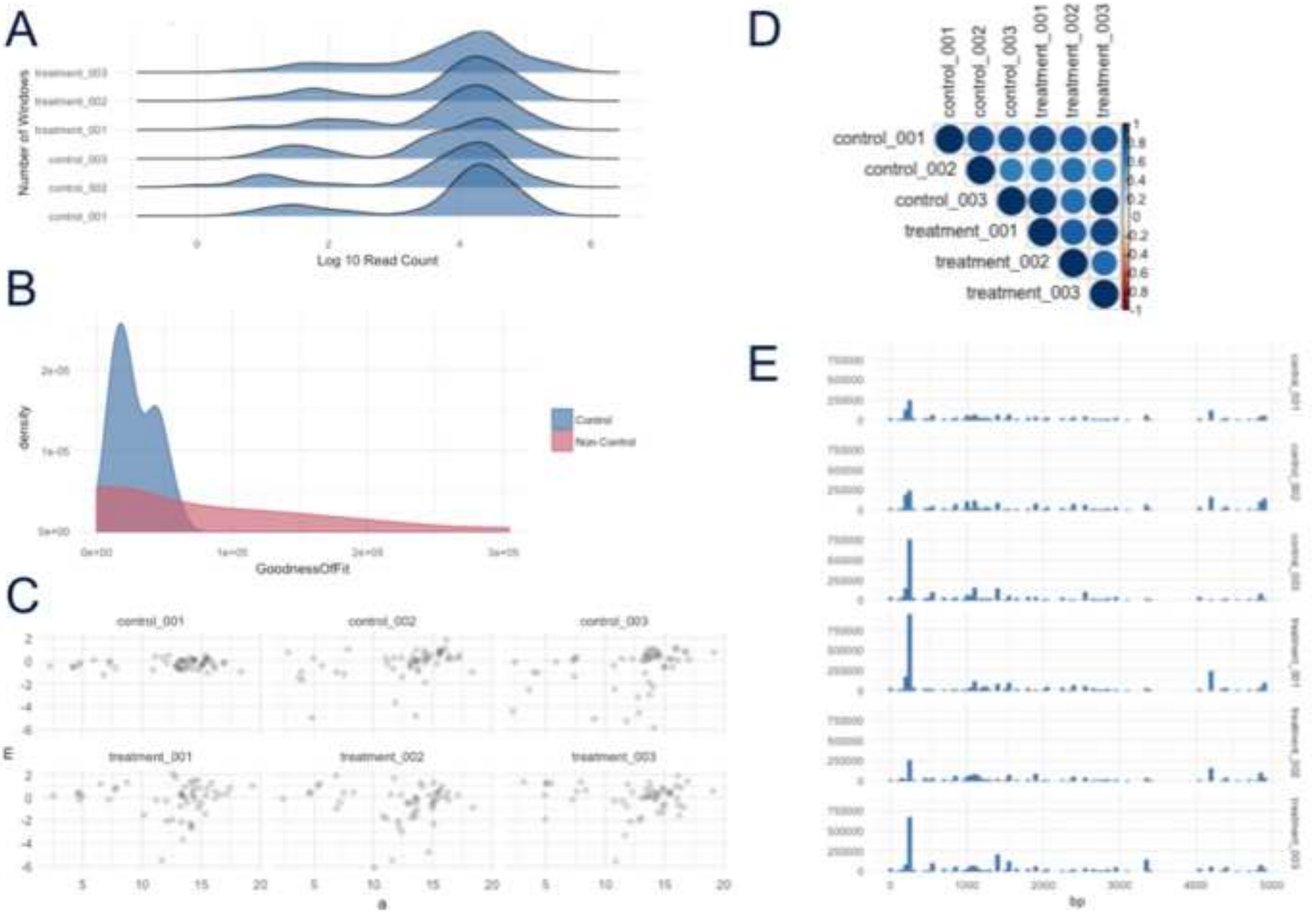

Manuscript

Click here to access/download
Supplementary Material
main.tex

Manuscript

Click here to access/download
Supplementary Material
gigascience-logo.pdf

Manuscript

Click here to access/download
Supplementary Material
main.bbl

Manuscript

Click here to access/download
Supplementary Material
ms.bib

Manuscript

Click here to access/download
Supplementary Material
oup-contemporary.cls

Manuscript

Click here to access/download
Supplementary Material
oup.pdf

Manuscript

Click here to access/download
Supplementary Material
vancouver-authoryear.bst

Click here to access/download
Supplementary Material
001_methods_comparison.html

Click here to access/download
Supplementary Material
001_methods_comparison.Rmd

Click here to access/download
Supplementary Material
002_methods_comparison.html

Click here to access/download
Supplementary Material
002_methods_comparison.Rmd

Click here to access/download
Supplementary Material
simulations.csv
