# Supplementary material for: A workflow for simplified analysis of ATAC-cap-seq data in R
Source: Gigascience. 2018 Jun 28;7(7):giy080. doi: 10.1093/gigascience/giy080 (PMC6047409; doi:10.1093/gigascience/giy080)
Supplement: Response_to_Reviewer_Comments_Original_Submission.pdf [file giy080_response_to_reviewer_comments_original_submission.pdf]

Author's Response To Reviewer Comments

Close

Dear Dr Zauner,

Thank you for taking the time to consider and have our manuscript reviewed. We were very pleased to receive your and the reviewer's positive and constructive comments. I am happy to be able to return our responses below. We have made all suggested changes and improvements to the software and manuscript and I hope you will find the changes satisfactory. Here is the requested point-by-point response to the comments.

Sincerely

Dan MacLean

Editor's comments

> In particular, I feel it is a valuable suggestion to provide real test data alongside the manuscript, to guide the reader from raw data to the final output with a real example dataset (referee 1's point #6, and similar point made by reviewer 2). We can host test data and other supporting material in our repository GigaDB. Our data curators would be happy to work with you to prepare a GigaDB dataset.

We have prepared a small, but real, ATAC-cap-seq data set that we are happy to share. It is from _Arabidopsis_ plants treated with a mock water treatment or a pathogen. The data are small enough that we can include them in the package itself and we have implemented a method that generates example input files that will load these data. By including the data in this way we allow a user to get started with the demo and tutorial easily.

> I also agree with the reviewers that it will be helpful for our readers if you provide a bit more background on the ATAC-cap-seq method and its use cases.

We have done this as described in the response to reviewer 2, by way of a new paragraph in the introduction section.

> In addition, please register any new software application in the SciCrunch.org database to receive a RRID (Research Resource Identification Initiative ID) number, and include this in your manuscript. This will facilitate tracking, reproducibility and re-use of your tool.

We have submitted at https://scicrunch.org/resources/about/registry/SCR_016286. The ID is included in the manuscript in the Supporting data and materials section.

Reviewer Comments

Reviewer 1

1. atacr plots correlations between samples as a QC measure. It would be useful if a clustering or PCA plot was also provided so the user can more easily verify sample mismatches, effect of treatment and batch effects.

We have added a new function `pca_plot()` that does this. It is also added as point in the new tutorial.

2. The authors should clarify that the package doesn't allow for experimental designs more complex than control/treatment. For example, the edgeR exact test is only for single factor data.

We have included text describing this feature in the section _Differential abundance and comparisons_ it now reads: `These can be run in single factor manner on pairs of sample, or on all samples simultaneously with a common reference sample specified by the user`

3. The authors should provide guidance to when the different normalization methods should be used.

The section _Diagnostic plots and normalisations now expands on this. It reads

```atacR provides a small set of useful normalisation methods applicable to small sets of target windows or those in which the large proportion show the same change in differential accessibility. A straightforward library size normalisation is provided. For most ATAC purposes this will be underpowered, because the low number of windows or high proportions of changing windows will cause skew between samples. This method useful when the experiment has reasonably high counts (> 20 mean) and it is certain few windows (< 10\%) will display differential counts. The atacR package also implements a dynamic method based on estimating the Goodness of Fit (GoF) measure described in \cite{poissonseq}. This method calculates GoF, a window/gene level measure of variability across all samples and selects the windows with lowest GoF as the subset on which to normalise. It is fast, automatically finds the least varying and best features in the data to normalise with and does a reasonable job of between-sample normalisation. It is usually the best one to choose. It is particularly useful when it is not known whether many windows will be changing or just a few will be, as it should perform the same regardless. Further to library size and GoF a user-led method is provided in which control windows corresponding to regions of the genome not expected to show differential accessibility can be defined in a text file. This is passed to a normalisation function that uses differences in these windows between samples or treatments to scale whole experiment counts. For ease of use with other normalisation strategies, a set of custom normalisation factors can also be provided as a simple vector and used directly.```

There is also section in the tutorial document describing the use cases of the different normalisation options.

4. differential_windows.Rmd doesn't seem to have an example of how to use edgeR for differential window analysis. Is estimateDisp() used? edgeR was created with genome-wide data in mind, instead of data from a few sites. edgeR borrows information from other genes to estimate dispersion of read counts. With so few sites in an ATAC-cap-seq data set, this procedure is unlikely to make sense. The authors must explain how they are using edgeR and how they adapted it to analysis of a few sites.

This is now corrected. `estimateDisp()` is used as we do want to take advantage of the strength of edgeR
```

on data where only a few windows show differential counts. It is not correct to assume _all_ ATAC data sets will show most windows change, only that it is a very, very much more likely possiblity than in eg RNAseq data sets. Hence, edgeR is still powerful in these situations with ATAC-cap-seq data as our analysis in Figure 2 shows - edgeR outperforms the other methods so using `estimateDisp()` in an unmodified manner is appropriate.

5. The package would benefit from a single tutorial like the ones existing for several R packages (e.g. the edgeR and DESeq2 vignettes), instead of several different files.

6. The authors should include a real dataset with raw data, i.e. .bam and metadata files, especially for peer-review along with a single file tutorial with all the steps necessary to go from raw data to differential windows.

We have made a tutorial, which can be viewed at https://teammaclean.github.io/atacr and is part of the source package. This satisfies the reviewer and editor request for a tutorial on real data. We still include the shorter topic based vignettes as this is more in line with how an R user will expect help to be presented in their packages.

7. pg 1, ln 30: I suggest avoiding phrasing that inverts the logical flow of thought ("upstream ATAC-seq step").

This now reads `ATAC-cap-seq is a high-throughput sequencing method that combines ATAC-seq with targeted nucleic acid enrichment of precipitated DNA fragment.`

8. pg 1, ln 55: The authors cite the original ATAC-seq paper for ATAC-cap-seq. Was this method published? Can the authors cite papers that used ATAC-cap-seq?

The ATAC library preparation method is essentially the same as the original ATAC-seq paper (Buenrostro 2015), we have now described in more detail the ATAC-cap-seq process that elaborates on this. Essentially ATAC-cap-seq is this combined with a standard enrichment step, which is established enough to have commercial providers of reagents for it. As such it isn't a quantum leap forward in sequencing tech and there isn't really a definitive paper to cite for the combined aspect.

9. How is the bait information used? Are windows stitched? Are non-baited windows used? Are only baited regions reported as differential? The authors should provide a comparison of their window-based method with standard peak callers and provide screenshots of the peaks and differential windows identified with the different methods.

Depends on what you tell atacR the data is. The package tries to do the expected thing from the user's point of view. The atac-cap seq loading method assumes you're not interested particularly in gene features or similar and divides the bait region into windows of interest depending on parameters set in the loading function by the user, so this can be fixed width, consecutive windows or overlapping windows. AtacR delegates this to the widely used csaw package so its pretty standard. For RNAcap seq it assumes you're interested in the whole bait region, so takes each one as a single window. This is described in the documentation, vignettes and new tutorial.

Non-bait regions are a special subset of large intra-region windows (each one is one window of full length), stats for these regions are calculated for summaries, to make sure off target counts are low as they should be. They can be used in analysis if the user chooses. The user can set the 'which' argument for almost all analytical functions to include 'bait_windows', 'non_bait_windows' and 'whole_genome' and any other user defined set of windows by applying standard BioConductor IRanges filters to the RangedSummarizedExperiment objects underlying the count data structures. This is all described in the documents, vignettes and now tutorials.

We don't think comparing user defined windows with the predictions of peak callers will be very informative. We aren't trying to compare peak vs window based calling, which is done in other places for other data types. If we carried this out we may see differences in the positions of windows, but it would not be clear if it was due to weaknesses in the binding and selection of baits, or the lack of power in the Peak Callers on particular data set used. We are not trying to work out how good a particular set of baits or peak callers is. If we did we'd still have implemented a method for window based analysis of count data.

10. Recall and precision are swapped in Fig 2.

This is corrected.

11. Overall, I think the manuscript should better explain how atacr performs each step, including information in comments 2, 3, 4 and 9.

This has been addressed in comments 2,3,4 and 9.

12. Fig 1: control_003 and treatment_002 seem to have been swappped.

I can't see to what the reviewer is referring. The data in the figure are correct as far as I can see.

Reviewer 2

1. I would like to see more information about the ATAC-cap-seq assay included in the manuscript to better relate the analysis pipeline to the assay method... It would be nice if the method was more explicitly stated, as well as when and how the method would be more beneficial than alternatives (such as just doing ATAC-seq).

This is a bioinformatics analysis method manuscript, and as such we don't think it is appropriate or helpful to add many details about a biochemical protocol. Also the data we describe in the paper is simulated, though there is some real data in the tutorials and descriptions of a biochemical method could mislead the reader as to the nature of the analysis this manuscript describes. We have added some expanded description of the ATAC-cap-seq method in the introduction section. We have also added a sentence to the introduction to press home the advantages of Capture technologies. This now includes

```A typical ATAC-cap-seq may be done by beginning with an ATAC-seq library as described previously \citep{Buenrostro:2015be}. Next, small (~9 nt) indexed barcodes can be used to amplify the ATAC libraries, Fragments are size selected, e.g. using SageELF to enrich sequences between 300bp and 1.2kb to give a uniform size distribution for multiplexing samples and replicates. Baits are designed and synthesised as 120 nt single-strand RNA baits covalently bound to biotinylated magnetic beads. These can be used in sequence capture with the multiplexed ATAC libraries. Libraries are quality checked then sequenced. Capture-seq is a cost-effective alternative to expensive whole genome analysis. Scientists can focus on loci of interest and multiplex multiple samples and data types for the same sequencing cost as a single whole genome sample. ```

2. It might also be worth explicitly stating that a capture approach would be able to detect differences in signal at previously identified loci, but would not show that the chromatin is "open" per se unless target capture probes were tiled across a locus.

We have added the following to the end of the first paragraph of the introduction

`ATAC-cap-seq does not show that chromatin is open in general, unless baits are tiled deliberately across continuous wide regions.`

3. It would be nice, however, to see the authors test their software on data simulated from actual ATAC-seq libraries in addition to the RNA-cap-seq data they currently use.

We have added actual ATAC-cap-seq sample data to the package that can be worked through in the worked example tutorial. This should allow a user to inspect and get used to working with the data sets of this type.

Close
```
