# Supplementary material for: A workflow for simplified analysis of ATAC-cap-seq data in R
Source: Gigascience. 2018 Jun 28;7(7):giy080. doi: 10.1093/gigascience/giy080 (PMC6047409; doi:10.1093/gigascience/giy080)
Supplement: Response_to_Reviewer_Comments_Revision_1.pdf [file giy080_response_to_reviewer_comments_revision_1.pdf]

Author's Response To Reviewer Comments

Close

Dear Editor,

Thanks for giving us the opportunity to respond to the reviewers comments. I believe that I have addressed them all. A number of the comments this time were seemingly included as comments in general or that did not need any action in the manuscript. I have answered those as if they were suggestions for changes or comments on the work itself. I have pasted below a point by point response to the reviewer.

Many thanks for your work

Dan MacLean

Point-by-point response

> 1. I am familiar with capture experiments. However, it is customary and good scientific practice to cite previous papers that have used a given technique, particularly when publishing analysis methods for said techniques.
> I also understand that this is a software application manuscript, but usually, software is written to analyze data from existing experiments and therefore proper contextualization is needed.
> Aren't there any papers published employing ATAC-seq followed by capture? I am wondering if the authors are proposing this method? If so, this should be clear in the text. The way it's written, it seems like ATAC-cap-seq is an established technique that has been used elsewhere.
> This manuscript must properly contextualize this tool and the authors should therefore state that they are proposing this technique. The description of the procedure now provided to reviewer 2 seems adequate, but as there is literature about capture-seq techniques, they should be cited.

The reviewer is correct, existing methodology should be cited, but as we pointed out in the previous response there are no prior ATAC-cap-seq experiments. We have cited some capture-sequence experiments. We understand the direction of the reviewers comments and sympathise, but we dont feel it fair or balanced to suggest that our approach is not following proper scientific practice because we have not cited, when (as we have pointed out in the previous responses) no such specific citation exists.

The reviewer makes the point that "usually, software is written to analyze data from existing experiments", and we think the key word here is "usually". In a lot of experiments you do get a situation where a biologist runs ahead and generates a lot of data and usually this is done without considering how the data are to be analysed. It will be something farmed out to a bioinformatician/statistician to deal with as best as can be done given the often poor state of the experimental design. Experiments are better designed if proper consideration of how to analyse them is done at the design phase. We have tried to do that here hence our workflow for a data type that is not yet directly citable as an extant, peer-reviewed published data set. We have plenty of samples for this in hand, but these have not gone through the long process of completing the biological experiments and further the publishing process so cannot yet be cited directly. However, ATAC-seq is cited, Cap-seq is cited and are known enough such that the two types can be combined to simulate and prepare software to analyse with.

The reviewer creates something of a false dichotomy when they state "the authors should therefore state that they are proposing this technique.". Im not sure we are proposing the technique, it may not be published elsewhere, but there also isn't a real example of it and its biological application in this manuscript. Furthermore we don't believe its such a novel idea that it needs to be proposed in such a sense. Combination of DNA extracted from some sample and then enrichment is a trivial idea at this time. We analyse simulated data to assess a workflow for data of that type. I think we should be wary of implying priority trying to claim priority for ATAC-cap-seq when no physical experiment exists here.

> I suggest rewriting the 2 first paragraphs of the introduction:
> - do not mention ATAC-cap in the first paragraph. Replace ATAC-cap-seq with ATAC-seq in the first sentence.
> - start a new paragraph to describe capture (paragraph 2).
> - move "Capture-seq is a cost-effective alternative..." to the the new paragraph 2 above.
> - it should be kept in mind that while capture-seq experiments are useful to target small sequencing spaces, the user still needs to sequence the data. This means either sharing a lane with other users, which complicates logistics, or pooling several replicates and experiments, which also complicates logistics. There is also an upfront cost to purchase baits, which only makes sense if capturing large numbers of replicates or experiments.
> - as ATAC-cap-seq doesn't seem to have been used in any publications, it shouldn't be mentioned as if it is an existing method. Instead, it should be presented as a new possibility proposed by the authors and put in the context of other capture-seq methods. I would suggest something like "Similarly to other methods (refs, examples, etc), one could envision coupling ATAC-seq with capture..."
> - present the software

We have approached the re-contextulisation by re-wording the first sentence to read: "ATAC-cap-seq can be conceptualised as a combination of two pre-existing, widely-used methods: the high-throughput sequencing of DNA from targeted enrichment capture performed on DNA fragments obtained from prior Assay for Transposase-Accessible Chromatin (ATAC)". Furthermore we have added the following penultimate sentence to the end of the first paragraph "It is a trivial step to consider combining ATAC-seq and capture to use the advantages of each in a single experiment. However, doing so will raise new analytic concerns, discussed more fully below."

We have done the above rather than applying the suggested edit, the suggested edit boils down to removing the first sentence which very briefly summarises ATAC-cap-seq and jumping straight into describing the preliminary upstream ATAC-step. In our view the first sentence gives a very high overview of what is expanded on subsequently and avoids giving the impression that the main object is ATAC-seq The other suggested edits seem to follow the flow of the manuscript as it is anyway (describe atac-seq, new paragraph, describe capture, present software).

> 2. Can atacr be used for other capture data, for example ChIP-seq? Are there any parameters that are tuned for ATAC? Why did the authors choose to focus on ATAC?

In principal, yes, atacR could be used on ChIP-seq, but its probably not the most straightforward workflow for the beginning ChIP-seq analyst. A bench biologist trying to analyse ChIP-seq data would be best served by the numerous whole genome workflows that are avaialbe for that sort of data. The

advantage of atacR for reduced representation data is that it packages up the fiddly and code heavy subsetting and cross-referencing of the regions corresponding to the baits from the whole genome and makes it easy for the beginning user to work with particular reference to the regions of interest.

We developed atacR because it is the data problem we have in hand and for which we found a useable solution lacking. atacR helps bridge the gap between the ChIP-seq tools and the ATAC-cap-seq problem.

> 3. My comment about window stitching was in reference to tiling experiments with overlapping windows, such as the region chr1:244,889-249,963 in the atacr example data. It's now clear that atacr will not stitch consecutive windows that are all differential, but merely report multiple windows, even if they are redundant.

We are pleased that the clarification was good enough to explain the operation of atacR.

> 4. The comment above is related to my previous comment on a comparison between atacr and existing peak caller approaches, which wasn't about peak calling itself, but about the differential windows. In the "peak caller" approach, users usually first find peaks, overlap them across replicates and expand them, count reads and perform differential count comparison. One contiguous region will be reported as differential. In atacr, this same region could be reported as a number of small regions, depending on the size of the baits (and likely if used with ChIP of certain histone marks), hence my curiosity to see how the two approaches compare.

Thanks to the reviewer for clarifying the nature of the comment, which we believe we answered in the earlier response.

> 5. What data is being plotted in the PCA? Raw counts or log transformed? Normalized? Lack of normalization and raw counts could explain the poor grouping of the samples. Normalized, log transformed data should be used instead for exploration of the data.

It is not clear to what the reviewer is referring. We presume that the PCA is the _example_ of how to generate a PCA plot in the atacR documentation/help as no PCA is done or concluded from or referred to in the manuscript. With regard to the PCA in the help document - that PCA is done on non-normalised data, as is clear from the tutorial and the arguments to the function made therein. The didactic structure of the tutorial document makes it easier to introduce the function as a QC/investigatory function before we get to the thornier issue of normalisation. In any analysis the user is able
to run the PCA plot at any time, any number of times on any section of the data, so can easily do it before or after normalisation, according to what the user thinks is a good approach for their experiment.

> 6. The Goodness of fit normalization method relies on the existence of several windows that are invariable. What is the minimum number/proportion of control windows that the user should specify? Some recommendation should be provided so users can design their baits accordingly.

Goodness of Fit is more subtle than that. It actually bridges the gap between using an invariant set of windows approach (which is accomodated in atacR by an appropriate function as described in the manuscript and tutorial/documentation) and total-count based normalisation. GOF normalisation will find the _least_ variable windows within a threshold - it does not rely on an absolutely invariant set. The

algorithm for computing this set is dynamic and if the windows are too variable a good set of invariant windows will not be found. The windows that are deemed invariant can conceivably be different from sample to sample. The original PoissonSeq paper in which the GoF normalisation is developed is cited.

We do not think it is wise to recommend a proportion of windows for users to design their baits. There isn't enough data available for us to make such a calculation and every experimental system would be different. We would hate if a number we suggested on not very broad data became used. If an experimenter is to choose to use an invariant set then they should be responsible for determining the proportion needed based on the variability in their experimental setup.

> 7. As far as I know, edgeR requires raw counts and scaling factors instead of normalized counts. The authors should check whether their normalization doesn't violate edgeR assumptions.

This is correct. But we don't force users to put normalised data through edgeR. We provide two other functions that are suitable for the normalised data and provide edgeR for situations that are appropriate - when data have only a few expected changing windows. The analysis on data described in the manuscript do not use any normalisations.

> 8. Why is the term "gene" used (for example in the normalization vignette and in Figure 2)?

In this data, the baits correspond to genic regions. It is a simple mix-up in terms while writing and has been corrected.

> 9. Regarding my comment in submission 1, in Figure 1E, on the extreme left, the sample labeled as control_003 has a very tall bar, while the other 2 control samples have very low bars. Two treatment samples have high bars in the same location, so maybe this was a sample swap - although it could be variation in the data (in which case more samples would be needed to confirm this difference).

We have checked carefully and the data are correctly labelled, the reviewer is correct to point out these data are variable, their observation is sound. The figure displays sample data only that are not used in concluding anything biological or methodological in the manuscript.

Close
