# Supplementary material for: A workflow for simplified analysis of ATAC-cap-seq data in R
Source: Gigascience. 2018 Jun 28;7(7):giy080. doi: 10.1093/gigascience/giy080 (PMC6047409; doi:10.1093/gigascience/giy080)
Supplement: Response_to_Reviewer_Comments_Revision_2.pdf [file giy080_response_to_reviewer_comments_revision_2.pdf]

Author's Response To Reviewer Comments

Close

None.

Close
