# Supplementary material for: A workflow for simplified analysis of ATAC-cap-seq data in R
Source: Gigascience. 2018 Jun 28;7(7):giy080. doi: 10.1093/gigascience/giy080 (PMC6047409; doi:10.1093/gigascience/giy080)
Supplement: Reviewer_1_Report_(Original_Submission) -- Noboru Jo Sakabe — 1/29/2018 Reviewed [file giy080_reviewer_1_report_(original_submission).pdf]

Reviewer Report

Title: **A workflow for simplified analysis of ATAC-cap-seq data in R**

Version: **Original Submission** **Date:** 1/29/2018

Reviewer name: **Noboru Jo Sakabe**

Reviewer Comments to Author:

In this manuscript "A workflow for simplified analysis of ATAC-cap-seq data in R" by Shrestha et al, an R package (atacr) wrapping existing tools was created to analyze ATAC-cap-seq data. The package receives alignment data as well as bait and other sample information as input, creates an R object that encapsulates the experiment data, provides QC plots, performs normalization and differential read count analysis.1. atacr plots correlations between samples as a QC measure. It would be useful if a clustering or PCA plot was also provided so the user can more easily verify sample mismatches, effect of treatment and batch effects.2. The authors should clarify that the package doesn't allow for experimental designs more complex than control/treatment. For example, the edgeR exact test is only for single factor data.3. The authors should provide guidance to when the different normalization methods should be used.4. differential_windows.Rmd doesn't seem to have an example of how to use edgeR for differential window analysis. Is estimateDisp() used? edgeR was created with genome-wide data in mind, instead of data from a few sites. edgeR borrows information from other genes to estimate dispersion of read counts. With so few sites in an ATAC-cap-seq data set, this procedure is unlikely to make sense. The authors must explain how they are using edgeR and how they adapted it to analysis of a few sites.5. The package would benefit from a single tutorial like the ones existing for several R packages (e.g. the edgeR and DESeq2 vignettes), instead of several different files.6. The authors should include a real dataset with raw data, i.e. .bam and metadata files, especially for peer-review along with a single file tutorial with all the steps necessary to go from raw data to differential windows.7. pg 1, ln 30: I suggest avoiding phrasing that inverts the logical flow of thought ("upstream ATAC-seq step").8. pg 1, ln 55: The authors cite the original ATAC-seq paper for ATAC-cap-seq. Was this method published? Can the authors cite papers that used ATAC-cap-seq?9. How is the bait information used? Are windows stitched? Are non-baited windows used? Are only baited regions reported as differential? The authors should provide a comparison of their window-based method with standard peak callers and provide screenshots of the peaks and differential windows identified with the different methods.10. Recall and precision are swapped in Fig 2.11. Overall, I think the manuscript should better explain how atacr performs each step, including information in comments 2, 3, 4 and 9.12. Fig 1: control_003 and treatment_002 seem to have been swappped.

Level of Interest

Please indicate how interesting you found the manuscript: An article whose findings are important to those with closely related research interests

Quality of Written English

Please indicate the quality of language in the manuscript: Acceptable

Declaration of Competing Interests

Please complete a declaration of competing interests, considering the following questions:

- Have you in the past five years received reimbursements, fees, funding, or salary from an organisation that may in any way gain or lose financially from the publication of this manuscript, either now or in the future?
- Do you hold any stocks or shares in an organisation that may in any way gain or lose financially from the publication of this manuscript, either now or in the future?
- Do you hold or are you currently applying for any patents relating to the content of the manuscript?
- Have you received reimbursements, fees, funding, or salary from an organization that holds or has applied for patents relating to the content of the manuscript?
- Do you have any other financial competing interests?
- Do you have any non-financial competing interests in relation to this paper?

If you can answer no to all of the above, write 'I declare that I have no competing interests' below. If your reply is yes to any, please give details below.

I declare that I have no competing interests

I agree to the open peer review policy of the journal. I understand that my name will be included on my report to the authors and, if the manuscript is accepted for publication, my named report including any attachments I upload will be posted on the website along with the authors' responses. I agree for my report to be made available under an Open Access Creative Commons CC-BY license (http://creativecommons.org/licenses/by/4.0/). I understand that any comments which I do not wish to be included in my named report can be included as confidential comments to the editors, which will not be published.

I agree to the open peer review policy of the journal

To further support our reviewers, we have joined with Publons, where you can gain additional credit to further highlight your hard work (see: https://publons.com/journal/530/gigascience). On publication of this paper, your review will be automatically added to Publons, you can then choose whether or not to claim your Publons credit. I understand this statement. Yes
