# Supplementary material for: A workflow for simplified analysis of ATAC-cap-seq data in R
Source: Gigascience. 2018 Jun 28;7(7):giy080. doi: 10.1093/gigascience/giy080 (PMC6047409; doi:10.1093/gigascience/giy080)
Supplement: Reviewer_1_Report_(Revision_1) -- Noboru Jo Sakabe — 5/23/2018 Reviewed [file giy080_reviewer_1_report_(revision_1).pdf]

Reviewer Report

Title: **A workflow for simplified analysis of ATAC-cap-seq data in R**

Version: **Revision 1** **Date:** 5/23/2018

Reviewer name: **Noboru Jo Sakabe**

Reviewer Comments to Author:

This is the second submission of the manuscript "A workflow for simplified analysis of ATAC-cap-seq data in R" in which the authors describe a software program to analyze differential ATAC-seq that makes use of capture baits to select regions of interest. The authors incorporated most of the suggestions and criticism that I had and clarified issues, improving the manuscript. However, there are still some points I would like to comment on:

1. I am familiar with capture experiments. However, it is customary and good scientific practice to cite previous papers that have used a given technique, particularly when publishing analysis methods for said techniques.
I also understand that this is a software application manuscript, but usually, software is written to analyze data from existing experiments and therefore proper contextualization is needed.
Aren't there any papers published employing ATAC-seq followed by capture? I am wondering if the authors are proposing this method? If so, this should be clear in the text. The way it's written, it seems like ATAC-cap-seq is an established technique that has been used elsewhere.
This manuscript must properly contextualize this tool and the authors should therefore state that they are proposing this technique. The description of the procedure now provided to reviewer 2 seems adequate, but as there is literature about capture-seq techniques, they should be cited.

I suggest rewriting the 2 first paragraphs of the introduction:
- do not mention ATAC-cap in the first paragraph. Replace ATAC-cap-seq with ATAC-seq in the first sentence.
- start a new paragraph to describe capture (paragraph 2).
- move "Capture-seq is a cost-effective alternative..." to the the new paragraph 2 above.
- it should be kept in mind that while capture-seq experiments are useful to target small sequencing spaces, the user still needs to sequence the data. This means either sharing a lane with other users, which complicates logistics, or pooling several replicates and experiments, which also complicates logistics. There is also an upfront cost to purchase baits, which only makes sense if capturing large numbers of replicates or experiments.
- as ATAC-cap-seq doesn't seem to have been used in any publications, it shouldn't be mentioned as if it is an existing method. Instead, it should be presented as a new possibility proposed by the authors and put in the context of other capture-seq methods. I would suggest something like "Similarly to other methods (refs, examples, etc), one could envision coupling ATAC-seq with capture..."
- present the software

2. Can atacr be used for other capture data, for example ChIP-seq? Are there any parameters that are tuned for ATAC? Why did the authors choose to focus on ATAC?

3. My comment about window stitching was in reference to tiling experiments with overlapping windows, such as the region chr1:244,889-249,963 in the atacr example data. It's now clear that atacr will not stitch consecutive windows that are all differential, but merely report multiple windows, even if they are redundant.

4. The comment above is related to my previous comment on a comparison between atacr and existing peak

caller approaches, which wasn't about peak calling itself, but about the differential windows. In the "peak caller" approach, users usually first find peaks, overlap them across replicates and expand them, count reads and perform differential count comparison. One contiguous region will be reported as differential. In atacr, this same region could be reported as a number of small regions, depending on the size of the baits (and likely if used with ChIP of certain histone marks), hence my curiosity to see how the two approaches compare.

5. What data is being plotted in the PCA? Raw counts or log transformed? Normalized? Lack of normalization and raw counts could explain the poor grouping of the samples. Normalized, log transformed data should be used instead for exploration of the data.

6. The Goodness of fit normalization method relies on the existence of several windows that are invariable. What is the minimum number/proportion of control windows that the user should specify? Some recommendation should be provided so users can design their baits accordingly.

7. As far as I know, edgeR requires raw counts and scaling factors instead of normalized counts. The authors should check whether their normalization doesn't violate edgeR assumptions.

8. Why is the term "gene" used (for example in the normalization vignette and in Figure 2)?

9. Regarding my comment in submission 1, in Figure 1E, on the extreme left, the sample labeled as control_003 has a very tall bar, while the other 2 control samples have very low bars. Two treatment samples have high bars in the same location, so maybe this was a sample swap - although it could be variation in the data (in which case more samples would be needed to confirm this difference).

Level of Interest

Please indicate how interesting you found the manuscript: Choose an item.

Quality of Written English

Please indicate the quality of language in the manuscript: Choose an item.

Declaration of Competing Interests

Please complete a declaration of competing interests, considering the following questions:

- Have you in the past five years received reimbursements, fees, funding, or salary from an organisation that may in any way gain or lose financially from the publication of this manuscript, either now or in the future?
- Do you hold any stocks or shares in an organisation that may in any way gain or lose financially from the publication of this manuscript, either now or in the future?
- Do you hold or are you currently applying for any patents relating to the content of the manuscript?
- Have you received reimbursements, fees, funding, or salary from an organization that holds or has applied for patents relating to the content of the manuscript?
- Do you have any other financial competing interests?
- Do you have any non-financial competing interests in relation to this paper?

If you can answer no to all of the above, write 'I declare that I have no competing interests' below. If your reply is yes to any, please give details below.

I declare that I have no competing interests

I agree to the open peer review policy of the journal. I understand that my name will be included on my report to the authors and, if the manuscript is accepted for publication, my named report including any attachments I upload will be posted on the website along with the authors' responses. I agree for my report to be made available under an Open Access Creative Commons CC-BY license (http://creativecommons.org/licenses/by/4.0/). I understand that any comments which I do not wish to be included in my named report can be included as confidential comments to the editors, which will not be published.

I agree to the open peer review policy of the journal

To further support our reviewers, we have joined with Publons, where you can gain additional credit to further highlight your hard work (see: https://publons.com/journal/530/gigascience). On publication of this paper, your review will be automatically added to Publons, you can then choose whether or not to claim your Publons credit. I understand this statement. Yes
