# Supplementary material for: A workflow for simplified analysis of ATAC-cap-seq data in R
Source: Gigascience. 2018 Jun 28;7(7):giy080. doi: 10.1093/gigascience/giy080 (PMC6047409; doi:10.1093/gigascience/giy080)
Supplement: Reviewer_2_Report_(Original_Submission) -- James Lewis — 3/15/2018 Reviewed [file giy080_reviewer_2_report_(original_submission).pdf]

Reviewer Report

Title: **A workflow for simplified analysis of ATAC-cap-seq data in R**

Version: **Original Submission** **Date:** 3/15/2018

Reviewer name: **James Lewis**

Reviewer Comments to Author:

Shrestha et al. developed an R software package designed specifically for determining differential enrichment of ATAC-seq reads from target capture ATAC-seq data. Their package includes three different test options (the edgeR Fisher's exact test, a Bayes factor t-test, and a bootstrap t-test). The authors apply each test to a range of simulated datasets, and find that edgeR performs best with low replicate counts, while the Bayes factor and bootstrap tests perform better as replicate numbers and the number of differentially enriched loci increase.

I like the idea of the manuscript, and entirely agree with the authors that more method-specific analysis pipelines are needed to address specific biases, rather than relying on software designed for RNA-seq studies. I would like to see more information about the ATAC-cap-seq assay included in the manuscript to better relate the analysis pipeline to the assay method. A quick search didn't show any publications for ATAC-cap-seq and no citation is given. I am assuming the protocol uses a target capture approach to enrich for DNA fragments after tagmentation but prior to amplification, allowing the researcher to test specific loci at high sequencing depth and relatively low cost. It would be nice if the method was more explicitly stated, as well as when and how the method would be more beneficial than alternatives (such as just doing ATAC-seq). It might also be worth explicitly stating that a capture approach would be able to detect differences in signal at previously identified loci, but would not show that the chromatin is "open" per se unless target capture probes were tiled across a locus.

I have no concerns with the software package as described. Providing differential enrichment test options and results for each test on simulated data is great and allows researchers to use the most appropriate test for their dataset and project. It would be nice, however, to see the authors test their software on data simulated from actual ATAC-seq libraries in addition to the RNA-cap-seq data they currently use.

Level of Interest

Please indicate how interesting you found the manuscript: An article whose findings are important to those with closely related research interests

Quality of Written English

Please indicate the quality of language in the manuscript: Acceptable

Declaration of Competing Interests

Please complete a declaration of competing interests, considering the following questions:

- Have you in the past five years received reimbursements, fees, funding, or salary from an organisation that may in any way gain or lose financially from the publication of this manuscript, either now or in the future?

- Do you hold any stocks or shares in an organisation that may in any way gain or lose financially from the publication of this manuscript, either now or in the future?
- Do you hold or are you currently applying for any patents relating to the content of the manuscript?
- Have you received reimbursements, fees, funding, or salary from an organization that holds or has applied for patents relating to the content of the manuscript?
- Do you have any other financial competing interests?
- Do you have any non-financial competing interests in relation to this paper?

If you can answer no to all of the above, write 'I declare that I have no competing interests' below. If your reply is yes to any, please give details below.

I declare that I have no competing interests

I agree to the open peer review policy of the journal. I understand that my name will be included on my report to the authors and, if the manuscript is accepted for publication, my named report including any attachments I upload will be posted on the website along with the authors' responses. I agree for my report to be made available under an Open Access Creative Commons CC-BY license (http://creativecommons.org/licenses/by/4.0/). I understand that any comments which I do not wish to be included in my named report can be included as confidential comments to the editors, which will not be published.

I agree to the open peer review policy of the journal

To further support our reviewers, we have joined with Publons, where you can gain additional credit to further highlight your hard work (see: https://publons.com/journal/530/gigascience). On publication of this paper, your review will be automatically added to Publons, you can then choose whether or not to claim your Publons credit. I understand this statement. Yes
